# Effectiveness of a phone-based nurse monitoring assessment and intervention for chemotherapy-related toxicity: A randomized multicenter trial

**DOI:** 10.3389/fonc.2022.925366

**Published:** 2022-09-15

**Authors:** Andrea Antonuzzo, Carla Ida Ripamonti, Fausto Roila, Andrea Sbrana, Luca Galli, Guido Miccinesi, Enrico Sammarco, Alfredo Berruti, Deborah Coletta, Laura Velutti, Alessandra Fabi, Domenico Cristiano Corsi, Gabriella Mariani, Patricia Di Pede, Gian Paolo Spinelli, Daniele Santini, Fable Zustovich, Marco Gunnellini, Maura Rossi, Monica Giordano, Massimo Di Maio, Gianmauro Numico, Paolo Bossi

**Affiliations:** ^1^ UO Oncologia Medica 1 SSN Polo Oncologico, Azienda Ospedaliero Universitaria Pisana, Pisa, Italy; ^2^ Oncology-Supportive Care Unit, Department Medical Oncology & Haematology, Fondazione IRCCS, Istituto Nazionale dei Tumori, Milan, Italy; ^3^ SC Oncologia Medica, Azienda Ospedaliera Universitaria “S. Maria della Misericordia”, Perugia, Italy; ^4^ Servizio di Pneumo-Oncologia, Azienda Ospedaliero Universitaria Pisana, Pisa, Italy; ^5^ Clinical Epidemiology Unit, Istituto per lo Studio, la Prevenzione e la Rete Oncologica, Firenze, Italy; ^6^ SC Oncologia Medica, ASST Spedali Civili di Brescia, Brescia, Italy; ^7^ UOSD Oncologia, Policlinico Tor Vergata, Rome, Italy; ^8^ Medical Oncology and Hematology Unit - IRCCS Humanitas Research Hospital, Humanitas Cancer Center, Milan, Italy; ^9^ Divisione Oncologia Medica A, IFO Istituto per la Ricerca dei Tumori Regina Elena, Rome, Italy; ^10^ Ospedale San Giovani Calibita Fatebenefratelli – Isola Tiberina, Rome, Italy; ^11^ Medical Oncology Department, Fondazione IRCCS Istituto Nazionale dei Tumori, Milan, Italy; ^12^ Unitá Operativa di Oncologia Universitaria della Casa della Salute di Aprilia, UOC Oncologia Universitaria, Aprilia, Italy; ^13^ UOC Oncologia Medica, Campus Bio-Medico, Rome, Italy; ^14^ UOC Oncologia, AULSS 1 Dolomiti, Ospedale San Martino, Belluno, Italy; ^15^ UO Oncologia, Ospedale di Gubbio – Gualdo Tadino, Gubbio, Italy; ^16^ SC Oncologia, ASO SS. Antonio e Biagio e Cesare Arrigo, Alessandria, Italy; ^17^ Oncologia, Ospedale S. Anna, Como, Italy; ^18^ Dipartimento di Oncologia, Università di Torino, AO Ospedale Mauriziano, Turin, Italy; ^19^ Department of Medical Oncology, Santa Croce e Carle Hospital, Cuneo, Italy

**Keywords:** chemotherapy-related toxicities, patient-reported outcome measures, nurse telephone monitoring intervention, quality of life, randomized multicenter trial

## Abstract

**Purpose:**

Anticancer treatment-related toxicities can impact morbidity and mortality, hamper the administration of treatment, worsen the quality of life and increase the burden on the healthcare system. Therefore, their prompt identification is crucial. NICSO (Italian Network for Supportive Care in Cancer) conducted a nationwide randomized trial to evaluate the role of a planned, weekly phone-based nurse monitoring intervention to prevent and treat chemotherapy, targeted therapy- and immunotherapy-related toxicities. Here, we report the results from the chemotherapy arm.

**Methods:**

This was a nationwide, randomized, open-label trial conducted among 29 Italian centers (NCT04726020) involving adult patients with breast, colon, or lung cancer and a life expectancy ≥6 months receiving adjuvant chemotherapy. Patients received either a weekly nurse monitoring phone call and an educational leaflet reporting practical advice about prevention and treatment of toxicities (experimental group) or the educational leaflet only (control group).

**Results:**

The addition of a nurse monitoring intervention may help reduce time spent with severe toxicities (grade ≥3), particularly those less frequently reported in clinical practice, such as fatigue. When considering grade 1–2 AEs, times with mild/moderate diarrhea, mucositis, fatigue and pain were shorter in the experimental arm. Time spent without AEs was significantly longer in the experimental arms for all the toxicities. The requirement for special medical attention was comparable between groups.

**Conclusion:**

This study suggests the need for implementing a better system of toxicity assessment and management for patients treated with adjuvant chemotherapy to promote effective preventive and/or therapeutic intervention against these events.

## 1 Introduction

The management of anti-cancer treatment-related toxicities has a central importance in patients with cancer. Indeed, therapy-related toxicities impair morbidity and mortality, hamper the correct administration of therapy, worsen the quality of life (QoL), and increase the burden on the healthcare system (1, 2). Therefore, prompt identification of treatment-related toxicities is crucial. Although international guidelines suggest algorithms for the management of treatment-related toxicities (3–5), low adherence to the prevention and management of toxicities has been reported, with increased incidence and duration of adverse events (AEs) (3, 6–12).

Different studies suggested that patient empowerment, intended as the gain of greater control over actions affecting health, should be promoted to improve the management of AEs, also with the use of a patient-reported outcome (PRO) evaluation tools (PRO-CTCAE, Common Terminology Criteria for Adverse Events) (13–20). Other studies suggest an improvement of therapy-related AEs when they are managed directly by home care nursing programs, mobile phone-based monitoring, or intensified clinical pharmacological/pharmaceutical care (19–21). Furthermore, significant improvements in relation to oral mucositis, diarrhea, constipation, nausea, pain, fatigue and insomnia (p<0.05) following oral capecitabine were observed in a group of patients who received a home care program by a nurse in addition to the standard care for 18 weeks. Unplanned service utilization was also lower in these patients (p=0.02) (19). In another study, a mobile phone-based advanced symptom management system was able to support the management of symptoms in patients with lung, breast and colorectal cancer receiving chemotherapy, who reported a significantly lower fatigue (p=0.040) (20). Lastly, an intensified clinical pharmacological/pharmaceutical care, including medication management and structured patient counseling, was associated with a positive effect on the number of medication errors, patient treatment perception, and severe side effects in a group of patients treated with oral anticancer drugs (21).

Nevertheless, literature evidence on this topic remains limited and robust data from further randomized multicenter trials are needed to better characterize the role of continuous monitoring of treatment-related AEs in cancer patients.

NICSO (Network Italiano per le Cure di Supporto in Oncologia, Italian Network for Supportive Care in Cancer) conducted a nationwide randomized trial to evaluate the role of a planned, weekly phone-based nurse monitoring intervention, provided together with an information leaflet versus the leaflet alone, to prevent and treat chemotherapy-, targeted therapy- and immunotherapy-related toxicities. Here, we report the results from the chemotherapy arm, which only included patients treated in the adjuvant setting; the results of the other two arms will be published separately, as follow-up is still ongoing.

## 2 Patients and methods

### 2.1 Study design

A nationwide, randomized, open-label trial has been conducted to evaluate the role of a planned, continuous phone-based nurse monitoring intervention, provided together with an educational leaflet, to prevent and treat chemotherapy-, targeted therapy- and immunotherapy-related toxicities. Patients assigned to the control arm received standard care, solely using the same educational leaflet used in the experimental arm given by the treating oncologist. We report the results only from the chemotherapy arm in the present work, which was limited to an adjuvant setting. According to the standard duration of adjuvant chemotherapy, the study period of each patient was the fixed duration of this treatment (4–6 months). In the chemotherapy-treated group, patients’ accrual lasted 18 months, from the beginning of the trial in March 2018, and lasted 24 months.

A total of 29 Italian centers were involved. Ethical Committees of all centers approved the study protocol (NCT04726020), and patients signed informed consent before enrollment.

### 2.2 Patients

Adult patients with radically resected breast (stage I–III), colon or lung cancer (stage II–III), a life expectancy of ≥6 months, receiving adjuvant chemotherapy (anthracyclines and cyclophosphamide ± taxanes, oxaliplatin and fluoropyrimidines, and combination of platinum or its derivatives, respectively) and available for phone contacts were enrolled. All chemotherapy regimens were administered according to current guidelines for the site of disease and stage.

The main exclusion criteria were symptomatic brain metastases or any condition that might impair patients’ compliance to protocol procedures; neoadjuvant chemotherapy or chemotherapy administered concurrently with radiation; prior exposure to any systemic oncological treatment; participation in other clinical trials.

### 2.3 Study procedures

At baseline, all patients received standardized counseling about the recognition, self-evaluation, and management of treatment-related AEs by their oncologists, who were also involved in periodic meetings to ensure standardization of all procedures. An educational leaflet accompanied counseling (see below).

Patients were then randomly assigned, in a 1:1 ratio, to receive: (i) a weekly nurse monitoring phone call scheduled with the patient, together with the educational leaflet reporting practical advice about prevention and treatment of toxicities and details about operative aspects of phone calls (experimental group); or (ii) only the educational leaflet (control group).

During the study, 12 questions from the PRO-CTCAE (17) (see for the Italian-validated questionnaire) according to the most frequent subjective AEs occurring with chemotherapy were administered weekly to each patient. The patient self-completed this questionnaire with a weekly automatic monitoring call. The patient could indicate verbally or key in numbers the rate of toxicity for each symptom, which was recorded by a computer; therefore, the patients answered the questions without talking to anyone.

#### 2.3.1 Development of the information leaflet

The information leaflet was developed after several national training meetings in 2017 about supportive therapy. At least one oncologist from each participating center attended these events. During these meetings, several health professionals (physicians and nurses), with the support of a specialist board (oncologists, dermatologists, gastroenterologists, cardiologists and endocrinologists, pharmacologists, pharmacists, pain therapists), developed an information dashboard.

The leaflet focused on the systemic treatment and the expected toxicities (see **Supplementary Material** – information leaflet). This intervention, based on national and international guidelines (Associazione Italiana Oncologia Medica [AIOM; www.aiom.it]; National Comprehensive Cancer Network [NCCN; www.nccn.org]; Multinational Association for Supportive Care in Cancer [MASCC; www.mascc.org]; European Society for Medical Oncology [ESMO; www.esmo.org]; American Society of Clinical Oncology [ASCO; www.asco.org]), represents the standard for the prevention and management of therapy-related toxicities.

#### 2.3.2 Nurse monitoring

A centralized nurse team performed the monitoring interventions (Nurse Operation Center, held by Vree Health Italia, a certified patient engagement solution provider). Oncologists of the scientific committee trained the nurses at the beginning of the trial and then every 6 months.

The nurse monitoring intervention consisted of a weekly phone call, according to a protocol of both predefined and individualized questions based on the leaflet. The call aimed to evaluate toxicities and offered appropriate preemptive and therapeutic management, reinforcing the notions reported in the leaflet. The nurse tried to contact the patient a maximum of three times each week; in case of lack of answer, that week’s intervention was considered failed.

The possible actions that might arise during the phone call (codified in the nurse algorithm) could be (i) advice on the use of drugs and therapeutic/preventive actions; (ii) facilitation of the contact between the patient and his/her referral specialist or general practitioner in case of toxicities that did not need urgent intervention (>12 hours); (iii) in case of toxicities needing an urgent intervention (<12 hours), facilitation of the contact with the referral physician or, if the contact was not possible, advice to access to the hospital through the center-specific modalities; and (iv) in case of not deferrable urgencies, direct access to the emergency room.

The Nurse Operative Center sent a synthetic report to the referring physician at the end of each phone call.

### 2.4 Study evaluations

The study’s primary endpoint was the proportion of time with severe toxicities over the total time the patient remained in the study. We chose to indicate the proportion of time with toxicity instead of time with toxicity to correct for differing time on treatments between the two groups.

The toxicities included in the evaluation were: diarrhea, mucositis, fatigue, nausea, vomiting and pain, as assessed by the PRO-CTCAE according to severity, frequency, and interference with usual or daily activities and referred to the previous 7 days. These symptoms were chosen among those clinically relevant and more likely responsible for reducing treatment adherence and, consequently, a possible reduction of treatment benefit. The grade reporting by means of PRO-CTCAE is performed by means of a Likert scale (e.g. 0=never; 1= rarely; 2=sometimes; 3=often; 4= always). The incidence and duration of grade 1–2 toxicities, the number of emergency room accesses and unplanned specialist visits (including medical oncologists), the number of hospital admissions, and days spent in the hospital because of treatment-related toxicities (as a whole) were secondary endpoints.

### 2.5 Statistical analysis

Considering the lack of data from the literature on severe side effects duration related to the overall treatment time, but estimating a prevalence of about 15% of time spent with grade ≥3 toxicity, given a power of 0.9 and an alpha value of 0.05, a total of 207 patients were necessary for each arm to show a reduction from 15% to 5% of the proportion of days spent with a grade ≥3 toxicity. Data are presented as number and percentage. To investigate the differences between the two groups (nurse monitoring vs standard monitoring) the relative risk was calculated. The statistical significance level was set at 5% for all tests. All the analyses were performed using MedCalc^®^ Statistical Software version 20.111 (MedCalc Software Ltd, Ostend, Belgium).

Data were analyzed using descriptive statistics. Differences between the two groups (nurse monitoring vs standard monitoring) were investigated with the Chi-square test.

## 3 Results

### 3.1 Study patients

**Figure 1:**
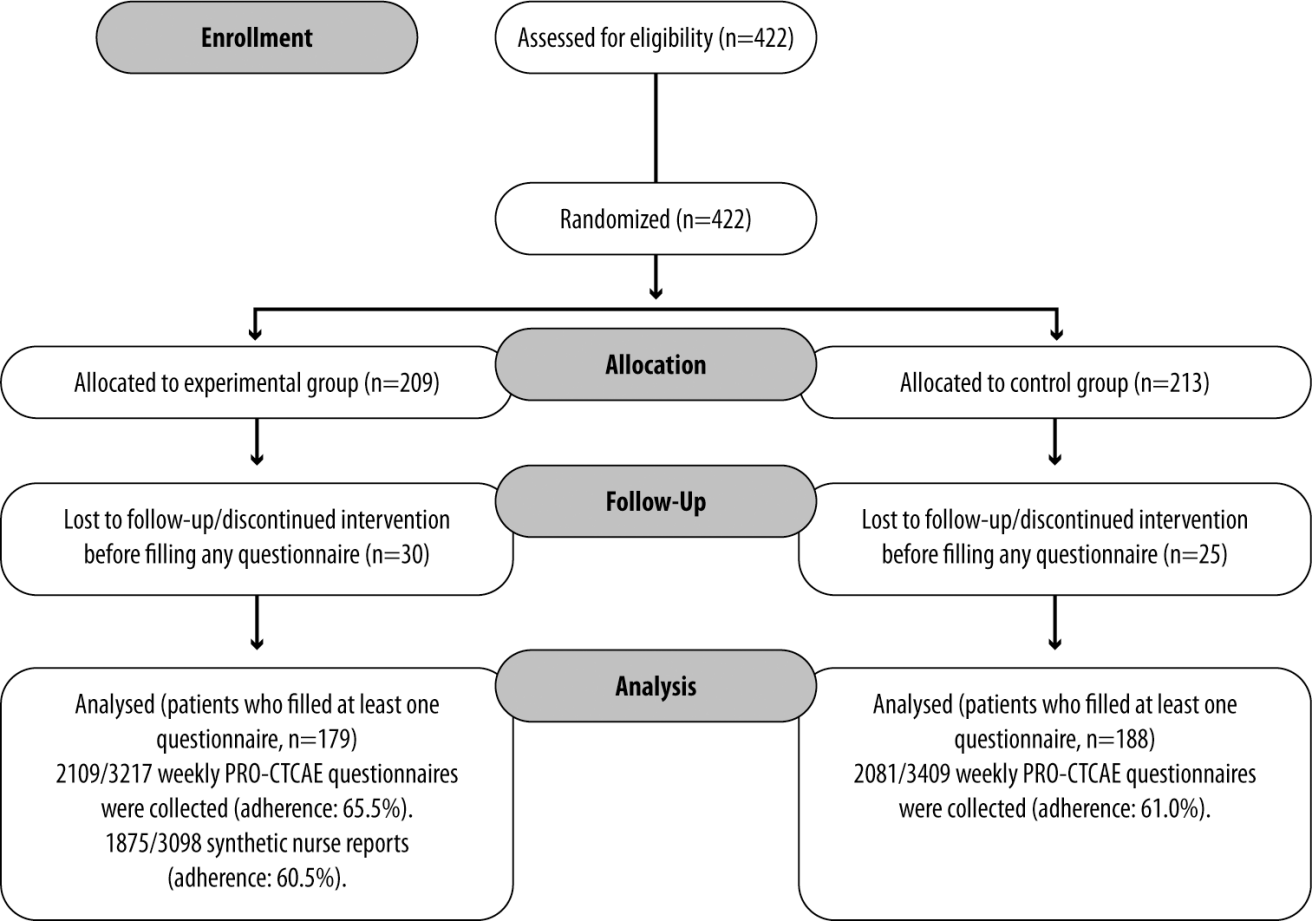
CONSORT diagram.

A total of 422 patients participated in the chemotherapy arm of the study: 209 patients were assigned to the experimental group and 213 to the control group (**Figure 1**). Patients who discontinued intervention before filling any questionnaire were excluded, while those who filled in at least one questionnaire were included. Baseline characteristics are reported in **Table 1**. More than two-thirds of patients were affected by breast cancer. The mean time spent in the study was 17 ± 5 weeks in the experimental group and 18 ± 6 weeks in the control group.

**Table 1:** Baseline characteristics and cancer diagnosis in the two groups.

	Experimental group (n=209)	Control group (n=213)
Sex: •Women, n (%) •Men, n (%) •Other, n (%)	161 (77) 48 (23) 0 (0)	160 (75) 53 (25) 0 (0)
Age (years), mean (SD) Age group: •0–40 years •40–50 years •50–60 years •60–70 years •Over 70 years	57 (12) 19 (9.1%) 57 (27.3%) 55 (26.3%) 56 (26.8%) 22 (10.5%)	59 (11) 12 (5.6%) 51 (23.9%) 58 (27.2%) 61 (28.6%) 31 (14.6%)
Diagnosis, n (%):		
•Breast cancer	140 (67)	153 (72)
•Colon cancer	62 (30)	56 (26)
•Lung cancer	7 (3)	4 (2)

After informed consent, 30 and 25 patients in the experimental and control group, respectively, did not continue the study due to a change in a therapeutic program, delay in chemotherapy initiation, informed consent withdrawal, or physician’s choice. In the experimental group, therefore, 179 patients were continuously evaluated. In the experimental group, 2109 weekly PRO-CTCAE questionnaires were collected out of 3217 expected, according to the permanence period in the study for each patient (adherence: 65.5%). A total of 1875 synthetic nurse reports out of 3098 were collected (adherence: 60.5%). Reasons for non-adherence to the nurse monitoring intervention were lack of answer to the nurse’s phone call or refusal of the patient to continue with the project.

In the control group, 188 patients completed the weekly PRO-CTCAE questionnaire, with 2081 collected questionnaires (3409 expected, adherence: 61.0%).

### 3.2 Time spent with toxicities

Overall, the percentage of time spent with any grade ≥3 toxicity was shorter in the experimental group compared with the control group (27.7% vs 30.9%; p ≤ 0.05). Time without AEs related to any toxicity was longer in the experimental group (14.4% vs 9.5%; p ≤ 0.001). No anti-cancer treatment-related deaths were reported.

Considering specific toxicities, a significant reduction in time spent with grade ≥3 fatigue (14.5% vs. 17.2%, p=0.015) and with grade 1–2 diarrhea (25.1% vs 28.3%, p=0.020), mucositis (22.1 vs. 25.8%, p=0.006), fatigue (60.6% vs 63.6%, p=0.047) and pain (36.4% vs 41.5%, p=0.001) was reported in the experimental group. Considering time spent without AEs related to specific toxicities, this was significantly longer in the experimental group: diarrhea (69.5% vs 65.2%, p=0.003), mucositis (75.2% vs 71.5%, p=0.008), fatigue (p<0.001), nausea (24.8% vs 19.1%, p=0.020), vomiting (90.6% vs. 88.1%, p=0.008) and pain (52.1% vs 47.1%, p=0.001) (**Table 2**).

**Table 2:** Time spent with or without AEs over the study period.

AEs	Time with G≥3 AEs (days)Incidence (%)	Time with G1–2 AEs (days)Incidence (%)	Time with any AE (days)Incidence (%)	Time without AEs (days)Incidence (%)
Any type:				
•Experimental group	585/2109 27.7*	1220/2109 57.8	1805/2109 85.6***	304/2109 14.4***
•Control group	643/2081 30.9	1240/2081 59.6	1883/2081 90.5	198/2081 9.5
Diarrhea:				
•Experimental group	113/2109 5.4	530/2109 25.1*	643/2109 30.5**	1466/2109 69.5**
•Control group	135/2081 6.5	589/2081 28.3	724/2081 34.8	1357/2081 65.2
Mucositis:				
•Experimental group	56/2109 2.7	467/2109 22.1**	523/2109 24.8**	1586/2109 75.2**
•Control group	56/2081 2.7	536/2081 25.8	592/2081 28.4	1489/2081 71.5
Fatigue:				
•Experimental group	306/2109 14.5*	1279/2109 60.6*	1585/2109 75.2***	524/2109 24.8***
•Control group	359/2081 17.2	1324/2081 63.6	1683/2081 80.9	398/2081 19.1
Nausea:				
•Experimental group	271/2109 12.8	670/2109 31.8	941/2109 44.6*	1168/2109 55.4*
•Control group	285/2081 13.7	718/2081 34.5	1003/2081 48.2	1078/2081 51.8
Vomiting:				
•Experimental group	33/2109 1.6	165/2109 7.8	198/2109 9.4**	1911/2109 90.6**
•Control group	50/2081 2.4	198/2081 9.5	248/2081 11.9	1833/2081 88.1
Pain:				
•Experimental group	241/2109 11.4	767/2109 36.4***	1008/2109 47.8***	1101/2109 52.2***
•Control group	237/2081 11.4	863/2081 41.5	1100/2081 52.9	981/2081 47.1

Experimental group: number of patients, 179, number of surveys, 2109.

Control group: number of patients, 188, number of surveys, 2081.

*p ≤ 0.05; **p ≤ 0.01; ***p ≤ 0.001.

Most frequently reported mild toxicities were fatigue (60.6% vs 63.6% of time; p≤0.05), pain (36.4% vs 41.5%; p≤0.001) and diarrhea (25.1% vs 28.3%; p≤0.05).

In patients with breast cancer, both time spent with mild toxicities (56.0% vs. 60.1%, p=0.024) and time without AEs (14.5% vs 9.0%, p<0.001) were significantly different in the experimental arm compared with controls, while in patients with colon cancer, only time with grade ≥3 toxicities was significantly shorter (21.4% vs 29.3%, p≤0.01; **Table S1**). Analysis of lung cancer was not performed due to the low number of patients (n=11).

### 3.3 Requirement for special medical attention

The number of unplanned outpatient service accesses, emergency room accesses, hospitalization, and unscheduled specialist visits was comparable between groups (**Table 3**). There was no statistically significant difference between the groups in all performed comparisons.

**Table 3:** Special medical attention over the study period.

	Experimental group	Control group
Access to the outpatient service, n (%) *Collected responses (n)	14 (4.0) 344	10 (2.8) 354
Emergency room access, n (%) *Collected responses (n)	10 (3.0) 343	7 (2.0) 354
Hospitalization, n (%) *Collected responses (n)	1 (0.3) 343	2 (0.6) 354
Specialist visits, n (%) *Collected responses (n)	23 (6.5) 344	23 (6.7) 356

## 4 Discussion

In this nationwide, randomized trial, we evaluated the impact of a predefined, weekly nurse-led telephone monitor on time spent with severe toxicities related to adjuvant chemotherapy. This intervention was added to standard counseling and the administration of an informative leaflet, which was the only support in the control group. Remarkably, a centralized nurse team led remote nurse monitoring to minimize the risk of bias and keep the management suggestions and patient empowerment as consistent as possible. The use of randomization also allowed to minimize any bias due to baseline characteristics of the enrolled patients.

Concerning grade ≥3 AEs, a significant time reduction with any severe toxicity was reported in the experimental group compared with the control group. When analyzing each type of grade ≥3 AEs, only time spent with severe fatigue was significantly shorter in the experimental group than in the control group. Mild toxicities (grade 1–2 AEs) can have a great daily impact in the patients’ QoL. Moreover, an anticipated detection and intervention of mild toxicities could prevent worsening of these AEs as well. Within this study, when considering grade 1–2 AEs, times with mild/moderate diarrhea, mucositis, fatigue, and pain were shorter in the experimental group. Of note, mild pain was the most reduced AE in the experimental group. Overall, the time spent without AEs was longer in the experimental arms for all the toxicities, especially for pain, suggesting that this kind of approach is helpful also to extend the period without AEs, with significant benefits to QoL.

These results can be explained as an effect of the weekly phone calls made by the nurses that allow the timely detection of toxicities often not reported in daily practice. This prompt evaluation can allow the institution of correct pharmacological treatment and thus reduce the time spent with that symptom. In addition, the phone call may have led to the prompt administration of practical advice to manage some toxicities, such as fatigue.

Therefore, the results reinforce existing data about the importance of the empowerment of patients and suggest that the nurse’s telephone intervention could result in better PROs and management of severe adjuvant chemotherapy-related toxicities (22–25).

The reduction of time spent with toxicities in the experimental group, although significant, was limited to a few percentual points and hence can be seen as poorly relevant from a clinical perspective. However, it should be considered that the nurse monitoring intervention might have prompted patients in the experimental group to more accurately describe their AEs. Thus, the benefit achieved by the phone call monitoring might have been underestimated.

Of note, we identified differences from expected in time spent with toxicities. According to our study, patients in the adjuvant setting spent most of their time experiencing some toxicities and about 30% of their time with grade 3–4 toxicities. On the other hand, time without experiencing any toxicity was limited to 10–15% of the total. This pattern was observed regardless of the type of toxicity or the initial diagnosis. This high rate could be explained by the self-reporting of toxicities by patients, while healthcare professionals may underestimate the rate of AEs (19).

At present, most trials on chemotherapy report only the frequency and severity of AEs. However, modern approaches for toxicity evaluation are also considering the time when analyzing treatment-induced AEs (26, 27). We have considered the time dimension and the symptom evaluation as directly reported by the patients through the analysis of PRO-CTCAE. This method increases the precision and the quality of the reporting and could better represent the burden of subjective toxicity induced by systemic treatments in the adjuvant setting.

Contrasting results were disclosed when analyzing the percentage of time spent with or without toxicities according to different primary tumors. In breast cancer, time spent without or with mild toxicities was shorter in the experimental arm, while in colon cancer – although data were collected on few patients – only time on grade ≥3 was significantly shorter. This suggests that the absence of toxicities may depend on drugs, schedules, type of patients and gender, thus warranting further research.

It is essential to put our trial within the framework of other studies. A systematic review on the safety of oral chemotherapy showed that educational interventions, remote telephone-based monitoring, and counseling improved the toxicity profile (28), although with no apparent effect on treatment adherence. The “AMBORA” trial recently showed a benefit of periodic counselling sessions in reducing AEs in patients on oral anti-cancer therapy (21). Similarly, another recent trial showed the efficacy of remote monitoring in preventing AEs in patients on adjuvant chemotherapy (29).

Our study presents some limitations. First, it is crucial to acknowledge that we have evaluated the presence of grade ≥3 AEs in a binary fashion (yes/no) in the last 7 days and not daily. Therefore, the actual duration of each event cannot be estimated with a precision of <7 days. Furthermore, adherence was <70% in both arms. Several reasons can have contributed to this suboptimal adherence. These include the long study duration for the patients, a high number of enrolling centers with a different commitment to the study, and the length of the accrual period that also involved part of the pandemic period. We should also acknowledge that we could not find differences in the two groups regarding ER access and unplanned visits; this could be the consequence of the limited power and the low numbers of these events in patients receiving adjuvant chemotherapy. The potential correlation of toxicity with study treatment was not evaluated during ER visits. Moreover, we should consider that most enrolled patients had breast cancer and were treated with anthracyclines and cyclophosphamide ± taxanes, therefore possibly influencing the broader application of the results to all adjuvant chemotherapy regimens. Only a few patients with lung cancer were enrolled, potentially leading to a bias in the grade of toxicities reported. Last, information on ECOG PS and comorbidities score were not collected, which may have led to an additional bias on the reported toxicities, especially for the elderly subgroup of patients.

Nevertheless, we believe that evaluating the duration of toxicities as the primary endpoint in our trial may be considered a suitable approach to get a more comprehensive description of adjuvant chemotherapy-associated AEs, as previously advocated (26, 30). Indeed, conventional toxicity tables usually include the incidence of high-grade AEs but do not provide information on the time course of these events. Instead, these latter have great importance because continuous, lower grade and durable toxicities are particularly relevant for long-term treatment tolerability and QoL impairment. We also advocate evaluating AEs duration in reporting the results of clinical trials.

In addition, we can suggest that this approach could be applied in selected populations of patients at higher risk of specific AEs, for instance, younger patients receiving chemotherapy, in whom some symptoms, such as fatigue, have a relevant impact on QoL (31, 32) or the opposite, elderly and frail patients, who could be at additional risk in case of severe toxicities.

## 5 Conclusion

The addition of a continuative, centralized nurse monitoring intervention may help reduce time spent with severe toxicities, particularly those less frequently reported in clinical practice, such as fatigue. A potential benefit of such a program could also deliver early medical intervention when needed.

This study, focused on a PRO, suggests the need for a call for action about the implementation of a better system of toxicity assessment and management for cancer patients treated with adjuvant chemotherapy, to provide more studies about the treatment of side effects, and to promote effective preventive and/or therapeutic intervention against these events. Data on targeted therapy and immunotherapy are being collected in the other arms of our trial to evaluate the role of the nurse monitoring in these settings.

## Data availability statement

The raw data supporting the conclusions of this article will be made available by the authors, without undue reservation.

## Ethics statement

The study protocol (NCT04726020) was approved by Ethical Committees of all centers. The patients/participants provided their written informed consent to participate in this study.

## Author contributions

Study conception and design: AA, PB, FR, CR. Collection and interpretation of data: all the authors. Statistical analysis: GMi. Manuscript drafting: AA, PB, CR, FR. Manuscript editing: all the authors. All authors contributed to the article and approved the submitted version

## Acknowledgments

Editorial, statistical and graphical assistance was provided by Simonetta Papa, PhD, Valentina Mirisola, Massimiliano Pianta, Valentina Attanasio and Aashni Shah (Polistudium SRL, Milan, Italy). This assistance was supported by internal funds.

## Conflict of interest

The authors declare that the research was conducted in the absence of any commercial or financial relationships that could be construed as a potential conflict of interest.

## Publisher’s note

All claims expressed in this article are solely those of the authors and do not necessarily represent those of their affiliated organizations, or those of the publisher, the editors and the reviewers. Any product that may be evaluated in this article, or claim that may be made by its manufacturer, is not guaranteed or endorsed by the publisher.
