# Supplementary material for: Effectiveness of a phone-based nurse monitoring assessment and intervention for chemotherapy-related toxicity: A randomized multicenter trial
Source: Front Oncol. 2022 Sep 15;12:925366. doi: 10.3389/fonc.2022.925366 (PMC9520968; doi:10.3389/fonc.2022.925366)
Supplement: Supplementary file 1 [file DataSheet_1.docx]

**Effectiveness of a phone-based nurse monitoring assessment and intervention for chemotherapy-related toxicity: a randomized multicenter trial**

**INFORMATION LEAFLET**

**FATIGUE**

**PREVENTION**

Practical tips

- If possible, eliminate or reduce causes that might induce fatigue (anemia, electrolyte disorders, metabolic disorder, nutrition disorders – such as anorexia – sleep problems, pain, psychological causes and lack of physical activity).

**TREATMENT**

Practical tips

- Organize your day to have enough time for rest and personal activities.
- Organize your housework throughout the entire week: it is better to do a little every day than a lot in one single day.
- Talk to your work manager if you are not able to maintain your normal rhythm of work
- Try to maintain your normal sleep patterns. Do not sleep more than necessary, just as much as you need to regain your strength.
- Several strategies can be used: muscle relaxation, meditation, yoga, stimulus-control therapies (i.e., associate bed only with sleep, not with tiredness, and try to regain a normal sleep pattern), sleep restriction techniques (i.e., stay in bed only for the time of sleep), but also the combination of several approaches.
- Waking up at the same time every morning is good to create a good sleep pattern. Try to avoid exciting substances (such as coffee, tea, and chocolate, especially in the hours before bedtime).
- Try and exercise regularly (150 minutes of physical activities per week or 30 minutes per day – walking, riding your bike, swimming).
- Try to have a correct food assumption (drink a lot, eat what you like, have small meals throughout the day) to have a good nutritional status and contrast weight loss, which reduces fatigue.

Drug therapy

- Methylphenidate: starting dose of 5 mg per os; increase the dose to 20 mg per day; if ineffective, stop the treatment.
- Dexamethasone (4 mg b.i.d.) or prednisone (12.5 mg b.i.d.) for 2 weeks, especially in patients with advanced disease.
- American ginseng (1.5–2 g b.i.d.).

**PAIN**

**PREVENTION**

Practical tips

- If possible, eliminate or reduce all the causes that might induce or prolong pain (wrong posture, sleepiness, anxiety, movement, weight laden on lesioned anatomic parts, incorrect or missed assumption of pain killers, assumption of pain killers on demand).
- Do not believe in fallacies about opioids since they are essential drugs for pain treatment and must be prescribed when needed.

**TREATMENT**

Practical tips

Keep a journal where you write down the intensity of your pain, the number of episodes of acute pain and their intensity, the pain killers you take, the benefit from their use, and possible side effects (e.g., nausea, vomiting, constipation and drowsiness).

Try to describe the type of pain you experienced (crampy, rodent, shooting, stinging, throbbing, compressive, tingling, aching, numbing).

Try to describe the site of pain, its duration, irradiation, triggering causes, relieving causes, interference with sleep, appetite, anxiety, depression, daily activities and quality of life.

All the data you collected will be fundamental for a correct pain evaluation and will be helpful to understand the best pain therapy to prescribe and/or how to change yours.

If the pain persists despite a correct assumption of pain therapy, it is important to contact the physician to anticipate your planned visit.

**Drug therapy**

- Pain should be cured and prevented; drugs should be prescribed “around the clock,” not only when needed. The drug’s dosing schedule should consider the type of used drug, its way of administration, its half-life, and its duration of action.
- Pain therapy depends on the pain intensity and the type of reported pain, and the efficacy of previous therapies.
- Acetaminophen and non-steroidal anti-inflammatory drugs (NSAIDs) are commonly used for mild pain. NSAIDs should be used for a limited time because of possible side effects on the stomach and kidney and only when there are no contraindications.
- The treatment of mild-to-moderate pain should be based on weak opioids, such as codeine and tramadol, or low-dose strong opioids, such as oral morphine, alone or in association with non-opioid drugs, such as acetaminophen or NSAIDs.
- Pain therapy in patients with moderate-to-severe pain is based on the use of strong opioids (morphine, oxycodone, fentanyl, buprenorphine, hydromorphone, methadone, tapentadol). There are no substantial differences in terms of efficacy between strong opioids; one of them should be used as a first-choice drug in moderate-to-severe cancer pain.

Fentanyl and buprenorphine are available as transdermic patches (with a gradual drug release over time).

- If pain is described as sharp or associated with pins and needles (since related to the involvement of nervous structures), opioids should be combined with adjuvants, such as gabapentin, pregabalin, or duloxetine.
- In patients with well-controlled pain, breakthrough pain (BTP) can appear. It is an acute, severe, transient pain that needs appropriate treatment. Rapid-onset opioids (ROOs) can control this short-lasting, intense pain, such as sublingual, transmucosal, nasal-spray fentanyl, or subcutaneous or intravenous (i.v.) morphine.

The use of oral morphine is suggested in predictable BTP, which is when a particular procedure or action causes BTP. It should be administered 30 minutes before the painful event.

n.b. The physician must prescribe the type of drug and its dosage according to the patient’s pain characteristics and clinical conditions.

**SIDE EFFECTS OF PAIN MEDICATIONS**

The most frequent side effect of opioids is constipation, which is often helped by reducing movement, poor hydration and/or food assumption.

The best treatment strategy is prevention. The correct treatment is based on addressing all possible causes, the withdrawal of possibly-related drugs that are not essential for the patient, the increase of liquid intake (when possible), the assumption of dietary fibers, and an adequate daily exercise (if the pain is well controlled), and the use of laxatives.

Laxatives can act with diverse mechanisms of action and are then often used in combination to boost up their effect in non-responsive patients. There is no evidence of the higher efficacy of some laxatives compared to others. The association of laxatives with different mechanisms of action is probably the most effective strategy. The clinical response, the patient’s compliance and preferences should drive the therapeutic choice.

A further strategy for opioid-induced constipation, when other laxatives are not effective, is based on naloxegol (pegylated naloxone), 12.5 mg or 25 mg per day. The standard dosage of 25 mg per day is associated with decreased bowel transit time with no effect on pain control. In contrast, the reduced dosage of 12.5 mg per day is suggested in patients with particular clinical conditions, such as kidney failure.

In the first period of opioid treatment, or when dosages are increased, some other side effects can come up, such as nausea, vomiting or drowsiness. These side effects are usually transient, they disappear within some days, and their intensity is highly variable among patients.

The use of drugs that can prevent or reduce opioid-induced side effects is mandatory to offer a better anti-pain treatment.

If pain and/or side effects persist despite the correct therapy, please contact the referral physician.

**NAUSEA and VOMITING**

**PREVENTION**

Practical tips

- Food: choose light food; avoid fried or spicy food. Eat small meals frequently during the day. Chew appropriately before swallowing. Drink a lot, but drink small amounts of water with small sips. Avoid drinking large amounts of water before eating. Eat neither too cold nor too hot food. Suggested beverages include coke, ginseng-based drinks, and chamomile. Suggested food in case of nausea includes bread, crackers, rusks, potatoes, bananas, and cereals.
- Do not dwell on thoughts related to nausea and vomiting.
- Take antiemetics as prescribed according to guidelines to avoid nausea and vomiting onset.

**TREATMENT**

Practical tips

- Ask someone to cook if the food smell is annoying or causes you nausea and/or vomiting.
- Avoid being near people who smoke.
- If you vomit, do not take food or oral drugs. Try and evaluate if you need further treatment (apart from around-the-clock drugs).
- If you vomit, try to take small sips of water or unflavoured beverages (water is the best option, but herbal tea or other overmentioned beverages can be used).

Drug tips

- Follow your home prescription as suggested by your physician, as briefly summed up by the following tables.


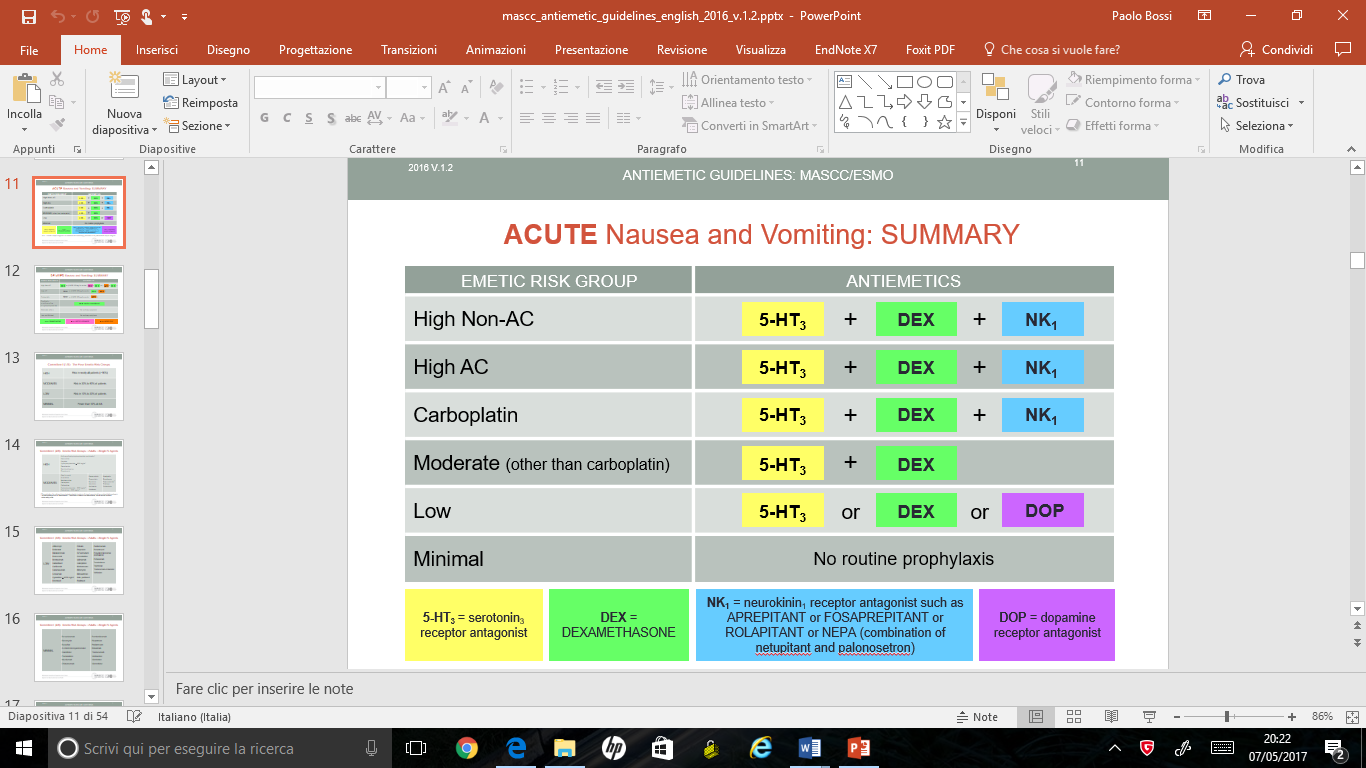


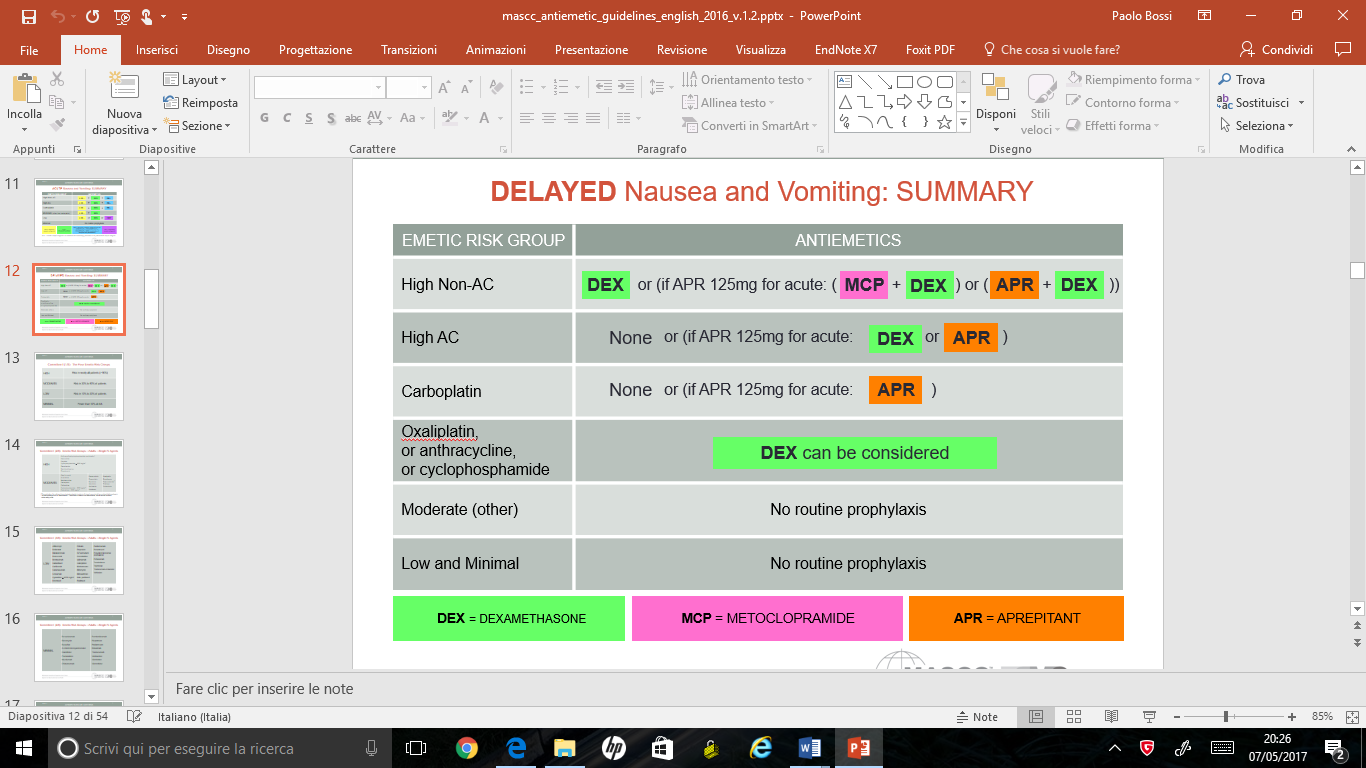


Therapy in case of nausea and/or vomiting that is not controlled despite correct prophylaxis.

- Olanzapine (oral drug, 5 mg per day, for up to 4–5 days). This treatment is off-label, but it is a unique drug with a proven efficacy from a randomized clinical trial.
- As an alternative, metoclopramide 10 mg intramuscular (i.m.) for up to 3-times per day in case of severe nausea and/or vomiting; or metoclopramide 10 mg per os, just before eating, in case of mild nausea and/or vomiting.
- If it is ineffective, dexamethasone 4–8 mg i.m.
- If severe nausea and/or vomiting lasts more than 2 days, please contact your referring physician.

**DIARRHEA**

**PREVENTION**

Practical tips

- Food: avoid food with a high lactose content (milk, fresh cheese), coffee, sparkling beverages, fat, fried or spicy food, legumes, sweet food, and chewing gum.

**TREATMENT**

Practical tips

- It is important to drink a lot. To avoid dehydration, it is helpful to eat food with a high content of potassium and sodium (such as vegetable soups) or soluble fibers (such as rice or oatmeal).
- Pay attention to personal hygiene, particularly washing the anal area with fresh water to avoid local irritation or infections.

Drug tips

- After the first discharge: loperamide 4 mg (two 2-mg tablets), then another 2-mg tablet after every discharge (maximum daily dose 16 mg, i.e., 8 tablets).
- If there is no improvement within 24 hours, diosmectite 3 g: 1–2 sachet after every discharge (maximum daily dose 3).
- If there is no improvement after another 24 hours, please contact your referring physician.

**IMMUNE CHECKPOINT INHIBITOR (ICI)-RELATED DIARRHEA AND/OR COLITIS**

**PREVENTION**

Practical tips

- Food: avoid food with a high lactose content (milk, fresh cheese), coffee, sparkling beverages, fat, fried or spicy food, legumes, sweet food, chewing gum.
- Basal evaluation of blood tests (complete blood count, liver and kidney function, LDH, electrolytes, TSH, fT3, cortisol, HBV, HCV, HBV-DNA when indicated, HIV, QuantiFERON).

**TREATMENT**

Practical tips

- Eat light food.
- Drink a lot. To avoid dehydration, it is helpful to eat food with a high content of potassium and sodium (such as vegetable soups) or soluble fibers (such as rice or oatmeal).
- Pay attention to personal hygiene, particularly washing the anal area with fresh water to avoid local irritation or infections.

*Pharmacological treatment (after exclusion of non-inflammatory causes) as per the following scheme:*

| **Grade**  ***NCI CTCAE v 4*** | **Definition** | **First-line treatment** | **Follow-up** |
| --- | --- | --- | --- |
| G1 | Less than four discharges per day  Asymptomatic colitis | Symptom treatment (e.g., loperamide, appropriate hydration)  **Continue with ICI** | A close clinical follow-up to evaluate symptom worsening.  Train the patient to look for timely contact in case of symptom worsening.  If worsening: treat as G2, G3 or G4. |
| G2 | 4-6 discharges per day; need of i.v. fluids for less than 24 hours; no interference with daily activities  Colitis with abdominal pain; bloody stool | Symptom treatment (e.g., loperamide, appropriate hydration)  **ICI should be temporarily interrupted** | If the condition goes back to G1, restart ICI.  If it lasts more than 48–72 hours:   - Start methylprednisolone 0.5–1 mg/kg/day i.v. or equivalent oral dosage - GI consultation - Evaluate abdomen–pelvis CT scan and colonoscopy - If the condition goes back to G1, taper steroids (at least 3 weeks of tapering), consider antibiotics for opportunistic infections and **restart ICI**   If it worsens or persists for more than 3–5 days with oral steroids, treat as G3–4 |
| G3-4 | More than 7 discharges per day, incontinence, need for i.v. fluids for more than 24 hours; interference with daily activities.  Colitis with severe abdominal pain that needs medical intervention; peritoneal signs  G4: life-threatening conditions; bowel perforation | **Stop ICI temporarily** (in case of G3 with PD-1/PD-L1 inhibitors) or **definitely** (in case of G3 with PD-1 + CTLA-4 inhibitors or G4)  **Hospital admission**  Stop oral food intake  Methylprednisolone 1–2 mg/kg/day (i.v.) or equivalent  Antibiotic prophylaxis for opportunistic infections  GI consultation  Colonoscopy  nb: in case of perforation immediate surgical consultation (avoid steroids, infliximab, antidiarrheal drugs) | If the condition gets better, keep on with steroids until G1, then a 1-month tapering  If it persists for more than 3–5 days or relapses after initial bettering, add infliximab 5 mg/kg (if not contro-indications), except for patients with bowel perforation and/or sepsis |

**CHEMOTHERAPY-INDUCED SKIN TOXICITY**

**Hand and foot syndrome (HFS)**

**PREVENTION**

*Practical tips*

- Train the patient to keep his/her skin appropriately hydrated
- Avoid trauma or infections
- Avoid hot or wet places
- Avoid the use of very hot water
- Avoid sun exposure
- Use cotton gloves, soft shoes and/or cushioned soles.

**TREATMENT**

*Practical tips*

- Stop and/or modify cancer drug dosage

*Drug treatment*

- Local medications
- Use of painkillers and/or cold packs

**Chemotherapy-induced nail toxicity**

**PREVENTION**

*Practical tips*

- Train the patient to have a good hand hygiene
- Train the patient to have his/her nails correctly cut (horizontal, parallel to nail basis)
- Avoid trauma and/or pressure
- Use cotton gloves and socks, soft shoes
- Avoid too acid detergents or toxic products

**TREATMENT**

*Practical tips*

- Stop and/or modify cancer therapy dosage

*Drug treatment*

- Local medications (it might include surgical removal of nails) and use of local antiseptics and/or antibiotics (especially if the pain is present)
- For periungual lesions (e.g., paronychia): local use of clobetasol if painless and no signs of infections; if infection signs, use of antiseptics (e.g., chlorhexidine, povidone-iodine) and/or antibiotics/antimycotics (after appropriate cultures)
- For periungual lesions, such as pyogenic granuloma: use of aluminium chloride-based astringent; local therapy with liquid nitrogen, steroids, or silver nitrate (surgery in case of refractory forms; phenolization if cryptonychia or ingrown nail)

**ANTI-EGFR SKIN TOXICITY**

**Acneiform rash (papulopustular rash)**

**PREVENTION**

*Practical tips*

- Train the patient to keep his/her skin well hydrated
- Avoid trauma and infections
- Avoid sun exposure

**TREATMENT**

*Practical tips*

- Remember that the intensity of the reaction is correlated with a good response to treatment (inform the patient)
- Stop and/or modify cancer treatment dosage according to the reaction severity

*Drug treatments*

- Oral tetracyclines (to start with the beginning of the treatment with anti-EGFRs, before the adverse event onset) (e.g., doxycycline 100 mg or minocycline 100 mg per day)
- Use of local antibiotics (clindamycin 1%, fusidic acid)
- Topical retinoids and/or benzoyl peroxide, even in combination (they can be irritative) or sulfosalicylic cream on a limited area (it can be irritative)
- If pruritus or severe inflammation, use of low-medium power steroid cream (e.g., denosine 0.05%, alclometasone 0.05%, fluocinonide 0.01%, 17-butyrate hydrocortisone 0.1%)
- If pruritus, consider water-based packs (with warm water), washes with rice starch, menthol-based cream

**Xerosis**

**PREVENTION**

*Practical tips*

- Moisturizer and sun cream

**TREATMENT**

*Practical tips*

- Use of detergent without foam

*Drug treatment*

- Moisturizing ointment or emollient cream

**Nail toxicity**

**PREVENTION**

*Practical tips*

- Train the patient to keep his/her hands and feet well hydrated
- Train the patient to a correct nail cut (parallel to the nail basis)
- Avoid trauma and/or pressures
- Use cotton gloves and socks, soft shoes
- Avoid contact with too aggressive detergents and toxic products

**TREATMENT**

*Practical tips*

- Stop and/or modify cancer drug treatment according to the toxicity severity
- Use detergents without foam

*Drug treatment*

- For periungual lesions (e.g., paronychia): local use of clobetasol if painless and no signs of infections; if infection signs, use of antiseptics (e.g., chlorhexidine, povidone-iodine) and/or antibiotics/antimycotics (after appropriate cultures)
- For periungual lesions like pyogenic granuloma: use of aluminium chloride-based astringent; local therapy with liquid nitrogen, steroids, or silver nitrate (surgery in case of refractory forms; phenolization if cryptonychia or ingrown nail)

**TYROSINE KINASE INHIBITOR SKIN TOXICITY**

**HFS and hyperkeratosis, desquamation, bullous lesions**

**PREVENTION**

*Practical tips*

- Train the patient to keep his/her hands and feet well hydrated
- Avoid trauma and/or infections
- Avoid hot or wet places
- Avoid the use of too hot water
- Avoid sun exposure
- Use cotton gloves and socks, soft shoes and/or cushioned soles.

**TREATMENT**

*Drug treatment*

*Grade 1*

- Use urea-based cream (urea 20%) b.i.d
- If there is pain or inflammation, use a powerful topic steroid
- Consider vitamin E supplementation (tocopherol 100–400 mg per day)

*Grade 2*

- Add painkiller (e.g., codeine)

*Grade 3*

- Same therapy as grade 2, but stop TKI

**IMMUNE CHECKPOINT INHIBITOR (ICI)-RELATED SKIN TOXICITY**

**Erythematous rash or maculopapular pruritus-associated rash**

**PREVENTION**

*Practical tips*

- Use delicate emollients
- Avoid sun exposure and use sun creams
- Avoid tight dressings or shoes
- Avoid scratching or scrubbing
- Basal evaluation of blood tests (complete blood count, liver and kidney function, LDH, electrolytes, TSH, fT3, cortisol, HBV, HCV, HBV-DNA when indicated, HIV, QuantiFERON).

**TREATMENT**

First, exclude non-inflammatory causes.

If excluded, use the following scheme:

| **Grade**  **NCI CTCAE v 4** | **Definition** | **First-line therapy** | **Follow-up** |
| --- | --- | --- | --- |
| G1–2 | Rash on <30% of the body surface, moderate pruritus | Supportive care  Evaluate local steroids  Antihistamine drugs  **Continue ICI** | If it lasts >12 weeks:   - Evaluate skin biopsy - Temporary discontinuation of ICI - Oral or i.v. methylprednisolone (0.5–1 mg/kg/day) with a slow tapering (at least 4 weeks) - Use antibiotic prophylaxis (opportunistic infections)   If worsening:  Treat as G3–G4 |
| G3–4 | >30% of body surface; reduction of capability of personal care or sleep interference  Life-threatening | **Temporary (G3) or definitive (G4) ICI discontinuation**  Evaluate skin biopsy  Dermatologist consultation  i.v. methylprednisolone 1–2 mg/kg/day or equivalent | If it gets better (G1):   - Steroid tapering (at least 4 weeks) and antibiotic prophylaxis for opportunistic infections - If indicated, restart ICI |

**ITCHING (pruritus)**

**PREVENTION**

*Practical tips*

- It is useful to drink a lot and have healthy food.
- It is important to keep your skin hydrated through the use of emollients.
- Wash daily with no aggressive detergents without foam
- Exclude other causes of pruritus (e.g., skin diseases or systemic diseases)

**TREATMENT**

*Practical tips*

- Use menthol moisturizers (even several times per day)

*Drug treatment*

*For localized itching:*

- Use antihistamine cream (e.g., dexchlorpheniramine) – consider the risk of contact-induced sensitization
- If no benefit is observed, use local steroids (e.g., desoximetasone)
- If ineffective, use 2% lidocaine cream or 1% pramocaine cream – consider the risk of contact-induced sensitization

*For widespread itching:*

- If interfering with sleep or limiting daily activities: use a sedative antihistamine (e.g., diphenhydramine or hydroxyzine)
- If ineffective, use:
  - Antidepressants (e.g., paroxetine) or benzodiazepine (e.g., diazepam)
  - Steroids (limited use because of side effects)
  - NK1 antagonists (e.g., aprepitant 80 mg on days 1, 3 and 5 – off label)
- If ineffective, contact your referring physician after two days

**ANTI-VEGF OR ANTI-VEGFR-INDUCED ARTERIAL HYPERTENSION**

**PREVENTION**

Arterial hypertension is the main cardiovascular risk factor in the western population. It has been shown that cancer patients treated with anti-VEGF or anti-VEGFR drugs are more prone to developing arterial hypertension or, if already affected, uncontrolled arterial pressure values due to the ongoing therapy. Possible mechanisms for such worsening are ascribed to the fact that these drugs induce a reduced production of nitric oxide (a vasodilating endothelial factor), a possible reduction of capillaries, and an increase in vascular system rigidity. It is then important that the patient start this class of drugs with the best pressure values according to the following practical tips.

*Practical tips*

- Try to control your bodyweight if overweight (BMI >25 kg/m^2^), reduce alcohol intake to 1 glass of wine per day, avoid smoking, limit stress conditions
- Reduce the use of salt (max 5 g of table salt per day), reduce the use of high-cholesterol food (reduce the use of food prepared with egg yolk, cheese, cold cuts – once a week; prefer fresh fish and avoid canned food that is plenty of salt- or fat-based preservatives)
- Do not overuse licorice and follow magnesium- and potassium-rich diet (cereals, fresh fruit, vegetables, citrus fruit)
- Avoid the use of painkillers, such as NSAIDs, or, if needed for pain control, use them after a physician’s prescription
- Have regular physical activities (e.g., walking, swimming, cycling).

Select patients with arterial hypertension and treat them according to clinical practice and European Guidelines of Arterial Hypertension

| **Category** | **Systolic** | **Diastolic** |
| --- | --- | --- |
| Optimal | <120 | <80 |
| Normal | 120–129 | 80–84 |
| Normal limit values | 130–139 | 85–89 |
| Grade 1 | 140–159 | 90–99 |
| Grade 2 | 160–179 | 100–109 |
| Grade 3 | ≥180 | ≥110 |
| Isolated systolic hypertension | ≥140 | <90 |

**During treatment**

*Practical tips*

The same practical tips as in the prevention phase are recommended.

It is to be considered that blood pressure values should be checked at least once a week during the first cycle of anti-VEGF therapy, then at least every 2–3 weeks during the treatment. Pay attention during the most common time frame (median onset time for bevacizumab 4.5–6 months, sorafenib 3 weeks, sunitinib 2 months, pazopanib 1 month). After the end of the first cycle and good blood pressure is obtained, a blood pressure check can be done at home and/or with GPs. The pressure goal is <140/90 mmHg for all patients on anti-VEGF therapy. Nonetheless, if the pressure target cannot be obtained, it is then not mandatory to postpone anti-VEGF therapy.

When to ask for a cardiologist consultation:

1. Before the start of treatment and at the onset of therapy-induced hypertension; if the patient is affected by ischaemic cardiopathy, heart failure, cardiomyopathy (dilatative, restrictive, hypertrophic), or heart arrhythmias
2. During the treatment, if the patient does not have an appropriate pressure value with the use of three drugs. In this case, it is also appropriate to stop cancer therapy.

***Drug therapy***

1. The first therapy treatment (aiming at using the least number of drugs) is an ACE inhibitor:

| **Drug** | **Dosage (available on the market)** |
| --- | --- |
| Enalapril | 5–20 mg |
| Ramipril | 2.5–5-10 mg |
| Perindopril | 5–10 mg |
| Lisinopril | 5–20 mg |

The increase of the dosages of ACE inhibitors or sartans (e.g., losartan, eprosartan, valsartan, irbesartan, candesartan, telmisartan – they are considered as effective as ACEi and used as a second choice in those patients experiencing side effects from ACEi) does not give a more powerful anti-hypertensive effect, but a longer duration of their action and then a better 24-hour effect. For this reason, after medical consultation, it is better to use the highest dosages in the above table. Always monitor sodium, potassium, and creatinine and stop treatment in case of kidney failure.

1. If ineffective, use an association with diuretics. On the market, it is possible to find pills containing both drugs (ACE inhibitor or sartan + diuretic).

| **Drug** | **Dosage (available on the market)** |
| --- | --- |
| Enalapril + hydrochlorothiazide | 20/12.5 mg |
| Ramipril + hydrochlorothiazide | 2.5/12.5 mg to 5/25 mg |
| Perindopril + indapamide | 5/1.25 mg to 10/2.5 mg |
| Lisinopril + hydrochlorothiazide | 20/12.5 mg |

These drugs need a daily administration, to be given preferentially in the morning. These combinations can induce a reduction in potassium or sodium (rarer). The use of diuretics can induce hyperuricemia.

3. If ineffective, consider using a **calcium antagonist** or **beta-blocker**.

| **Class** | **Drug** | **Dosages** |
| --- | --- | --- |
| Calcium antagonist | Amlodipine | 5–10 mg |
| β-blocker | Bisoprolol | 1.25–2.5–3.75–5–7.5–10 mg |
| β-blocker | Nebivolol | 5 mg |

Use daily administration.

Apply a drug titration.

**Calcium antagonists** are to be administered preferably in the morning (they can induce nocturia at night) and **β-blockers** (to avoid bradycardic effect at night). In case of withdrawal (especially for beta-blockers), apply dose tapering.

**MUCOSITIS**

1. Hygiene and oral care protocols must be used to prevent mucositis regardless of age and type of cancer treatment. It is recommended to have good oral hygiene by brushing teeth with a soft-bristle toothbrush 3-times a day.
2. Rinse with benzydamine for oral mucositis prevention or water with sodium bicarbonate or other mouthwash
3. 30-minute oral cryotherapy to prevent oral mucositis in patients receiving 5-fluorouracil bolus.
4. Use of appropriate pain therapy for mucositis: transdermal fentanyl effectively controls pain related to oral mucositis induced by chemotherapy or radiotherapy.
5. 2% morphine rinses can effectively treat pain due to oral mucositis in patients undergoing radiotherapy for head and neck neoplasms.
6. 0.5% doxepin rinses can be suggested in the treatment of stomatitis-induced pain

Nutrition in patients with mucositis must be as soft as possible, avoiding spicy or salty or too hot food.

To manage mucositis-induced pain, it is important to take a painkiller, such as oral morphine, 30 minutes before the main meals or rapid-onset fentanyl a few minutes before food.

**IMMUNOTHERAPY-INDUCED PNEUMONIA**

**BASAL EVALUATION**

- Basal evaluation of blood tests (complete blood count, liver and kidney function, LDH, electrolytes, TSH, fT3, cortisol, HBV, HCV, HBV-DNA when indicated, HIV, QuantiFERON).
- Exclude non-inflammatory causes. Exclude infective causes associated with similar presentation (diffuse/interstitial), such as viral pneumonitis or atypical organisms (Chlamydia, Mycoplasma).
- Exclude other drug-induced pneumonia.
- When suspected immunotherapy-induced pneumonia, perform respiratory function tests, arterial blood gas tests, and chest CT scans (or X-ray).

**TREATMENT**

See table.

| **Grade**  NCI CTCAE v 4 | **Definition** | **Management** | **Follow-up** |
| --- | --- | --- | --- |
| G1 | Imaging alteration; asymptomatic | Evaluate if postpone ICI  Monitor patient every 2–3 days for symptoms  Evaluate pneumologist and/or infectious disease specialist consultation | Re-evaluate with appropriate imaging at least after 3 weeks  If worsening, treat as G2 or G3–4 |
| G2 | Mild-to-moderate new symptoms | Postpone ICI  Pneumologist and/or infectious disease specialist consultation  Monitor the patient daily (evaluate if hospitalization is needed)  Start i.v. methylprednisolone 1 mg/kg/day or equivalent  Evaluate bronchoscopy or lung biopsy | Re-evaluate with imaging every 1–3 days  If it gets better:   - When symptoms are the same as basal condition, taper steroids (at least one month) and restart ICI; consider antibiotic prophylaxis   If there is no variation within 2 weeks or worsening:   - Treat as G3–4 |
| G3–4 | Severe new symptoms; hypoxia (onset or worsening); life-threatening | Permanently stop ICI  Hospitalization  Pneumologist and infectious disease specialist consultation  I.v. methylprednisolone 2–4 mg/kg/day or equivalent  Antibiotic prophylaxis  Evaluate bronchoscopy/lung biopsy | If it gets better:   - Taper steroids in at least 6 weeks   If there is no improvement within 48 hours:   - Add other immunosuppressors (es infliximab, cyclophosphamide, IVIG, mycophenolate mofetil) |

**IMMUNOTHERAPY-INDUCED RENAL FAILURE**

**BASAL EVALUATION**

- Basal evaluation of blood tests (complete blood count, liver and kidney function, LDH, electrolytes, TSH, fT3, cortisol, HBV, HCV, HBV-DNA when indicated, HIV, QuantiFERON).
- Exclude non-inflammatory causes

**TREATMENT**

| **Grade**  NCI CTCAE v 4 | **Definition** | **Management** | **Follow-up** |
| --- | --- | --- | --- |
| G1 | Creatinine ULN – 1.5 ULN | Continue ICI  Weekly monitoring of creatinine | If it gets better, keep on with standard monitoring  If worsening, treat as G2 or G3–4 |
| G2-3 | Creatinine 1.5–6 ULN | Postpone ICI  Monitor creatinine every 2–3 days  Start 0.5–1 mg/kg/day i.v. methylprednisolone or equivalent | If it goes back to G1:   - Taper steroid in 1 month - Evaluate antibiotic prophylaxis - Restart ICI and standard creatinine monitoring   If it lasts more than 7 days or worsens:   - Treat as G4 |
| G4 | Creatinine >6 ULN | Permanently stop ICI  Daily creatinine monitoring  Start methylprednisolone 1–2 mg/kg/day or equivalent  Nephrologist consultation  Evaluate renal biopsy | If it goes back to G1, taper steroid within 1 month and evaluates antibiotic prophylaxis |

**IMMUNOTHERAPY-INDUCED LIVER FUNCTION ALTERATIONS**

**BASAL EVALUATION**

- Basal evaluation of blood tests (complete blood count, liver and kidney function, LDH, electrolytes, TSH, fT3, cortisol, HBV, HCV, HBV-DNA when indicated, HIV, QuantiFERON).
- Exclude non-inflammatory causes.

**TREATMENT**

| **Grade**  **NCI CTCAE v 4** | **Definition** | **Management** | **Follow-up** |
| --- | --- | --- | --- |
| G1 | AST or ALT = ULN – 3 ULN and/or  Total bilirubin = ULN – 1.5 ULN | Continue ICI | Standard monitoring  If worsening, treat as G2 or G3–4 |
| G2 | AST or ALT = 3-5 ULN and/or  Total bilirubin = 1.5–3 ULN | Postpone ICI  Monitoring every 3 days | If it goes back to basal values:   - Restart standard monitoring - Restart ICI   If it persists more than 5–7 days or worsens:   - Start i.v. methylprednisolone 0.5–1 mg/kg/day or equivalent   If it goes back to G1 or basal, taper the steroid in one month. Consider antibiotic prophylaxis. Restart ICI. |
| G3-4 | AST or ALT >5 ULN and/or total bilirubin >3 ULN; life-threatening | Permanently stop ICI  Blood test monitoring every 1–2 days  Start i.v. methylprednisolone 1–2 mg/kg/day or equivalent  Antibiotic prophylaxis  GI consultation | If it goes back to G2, steroid tapering in 1 month  If it does not get better in 3–5 days or worsens or recurs:   - Add mycophenolate mofetil 1 g b.i.d. - If it does not get better in 3–5 days, add other immunosuppressors |

**IMMUNOTHERAPY-INDUCED ENDOCRINOPATHIES**

Basal evaluation of blood tests (complete blood count, liver and kidney function, LDH, electrolytes, TSH, fT3, cortisol, HBV, HCV, HBV-DNA when indicated, HIV, QuantiFERON).

Assessment

It is important to evaluate thyroid function before every administration of immunotherapy.

**An increase in TSH values can represent an EARLY BIOMARKER of the hypothalamus–hypophysis–thyroid axis alteration.**

**In the case of TSH increase, evaluate:**

- **Cortisol (8 a.m., fasting)**
- **ACTH**
- **LH and FSH**
- **Estradiol (women)**
- **Testosterone (men)**
- **Prolactin**
- **ADH (ipilimumab-induced diabetes insipidus)**

If hypophysitis is suspected, perform **skull base MRI**: possible observation of hypophysis enlargement (60-100% of volume enlargement) or hypophysis nodules (consider the possibility of pre-existing nodules)

**Cortisol evaluation**

Normal values **8–25 µg /dL**

- **<3 µg/dL** : hypoadrenalism
- **>18 µg/dL** : normal adrenal function
- Values between **3–18 µg/dL** (grey zone): dose ACTH to exclude if other evaluations are to be made (e.g., subclinical hypoadrenalism to monitor)

Attention

***In case of concomitant steroid therapy**, evaluate cortisolemia in the morning before taking medication. It is better not to take steroid medication the afternoon before the evaluation.

**THERAPY**

**Thyroid alteration**

**Hyperthyroidism:** β-blocker (es. propranolol 40 mg: ½ capsule three times per day if heart rate <100 bpm or 1 capsule 3-times per day if heart rate >100 bpm);

**Hypothyroidism:** start Levothyroxine (L-T4) in symptomatic patients

- TSH >50: L-T4 2 µg/kg/day
- TSH <50: L-T4 1 µg/kg/day

In older patients or patients with cardiac disorders: start with 25 µg/day and increase the dosage every week until 1–2 µg/kg/day (risk of ischaemic cardiopathy).

Hypothyroidism can be corrected, and ICI can be continued. It is important to assess if hypothyroidism is primary (high TSH with low fT4) or secondary to hypophysitis (low TSH with low fT4).

**A mild TSH increase (<10 UI/mL) with no symptoms does not need therapy.**

**Hypopituitarism**

- In the case of asymptomatic hypopituitarism:

Start steroid therapy at least one week before L-T4 treatment starts

- In case of symptomatic hypopituitarism (headache, fatigue, eye disorders, hypotension, imaging evidence of hypophysis enlargement), it is important to assess low ACTH or TSH values and start methylprednisolone 1–2 mg/kg per os or i.v. (if hypotension)
- After 2–4 weeks, start hormone replacement therapy (L-T4, estrogen or testosterone, ADH, etc) according to the presence of hormonal deficits.

**Adrenalitis**

**Adrenal failure**

- Primary: low cortisol, high ACTH (primary adrenal disorder)
- Secondary: low cortisol, low ACTH (primary hypophysis disorder)

**Acute adrenal failure (adrenal crisis)**

- Assessment and intervention must be rapid
- I.v. hydrocortisone: 100 µg/6 hours for 24 hours

If it gets better:

I.v. hydrocortisone: 50 µg/6 hours (maintenance therapy to start after 3–4 days)

Add fludrocortisone (mineralocorticoid) if needed

- Correct volume depletion, dehydration, and hypoglycemia with NaCl solution and glucose

Evaluate and treat infections and other precipitating factors

| **Table S1. Percentage of time spent with or without AEs over the study period according to the type of cancer diagnosis.** | | | | | |
| --- | --- | --- | --- | --- | --- |
| **Group** | **Number of patients** | **Number of surveys** | **Time with G≥3 AEs (%)** | **Time with G1–2 AEs (%)** | **Time without AE (%)** |
| **Breast cancer** | | | | | |
| Experimental group | 128 | 1436 | 29.5 | **56.0*** | **14.5***** |
| Control group | 136 | 1526 | 31.0 | 60.1 | 9.0 |
| **Colon cancer** | | | | | |
| Experimental group | 55 | 649 | **21.4**** | 64.1 | 14.5 |
| Control group | 48 | 501 | 29.3 | 58.7 | 12.0 |
| Statistically significant differences are reported in bold. *p≤0.05; **p≤0.01; ***p≤0.001. | | | | | |
